# Supplementary material for: Gene name errors: Lessons not learned
Source: PLoS Comput Biol. 2021 Jul 30;17(7):e1008984. doi: 10.1371/journal.pcbi.1008984 (PMC8357140; doi:10.1371/journal.pcbi.1008984)
Supplement: Attachment — Submitted filename: GeneNames_Point-by-point response to reviewer.docx [file pcbi.1008984.s005.docx]

# Point-by-point response to reviewers

## Reviewer #1

**The authors have written a clearly readable paper with all relevant data and code made available.**

**It is beneficial to highlight how incorrect gene names continue to be published and how this can be an issue as they are difficult to check for.**

We thank reviewer 1 for their positive feedback and constructive criticism.

**R1P1. It is unclear how useful the comparison between different publications is.**

Response: Not sure which publication is referred to here so I will address all:

Here “publications” means this and the gene name error article from 2016. Some readers may remember the headline from the 2016 article that “approximately one-fifth of papers with supplementary Excel gene lists contain erroneous gene name conversions”. They may be surprised that now suddenly this figure is closer to 30%. Therefore we have outlined the major contributors to this discrepancy in the Discussion (page 16).

Here “publications” means the three selected mega-journals: Nature Comms, PLOS ONE and Scientific Reports. We selected these journals for comparison because they publish a high proportion of articles in biomedical science and genomics. Editorial systems put in place in these journals are likely to affect the aggregate quality and reliability of the literature as a whole. The unique data requirements of Nature Communications are mentioned in the Discussion (page 17) is cited as the main contributor for why this is the #1 journal for gene name errors.

**R1P2. It is unclear how many other users will see value in the provided software.**

Response: The scripts provided in the linked GitHub repository (page 24) are provided for the purpose of reproducibility. For the benefit of other researchers who would like to undertake similar research and build on top of our work.

**R1P3. It is also unclear whether it was necessary to spend time focusing on which organisms have gene names at risk, and the text devoted to gene name errors by year.**

Response: We have included this section to highlight to readers that there are still many gene names at risk of date conversion. Despite changes being made for human and mouse genes [see ref 3], these are yet to propagate to many other species in Ensembl. For the reader’s benefit, we have added additional context to this section by making the following change (top of page 6):

“Although recent changes have been made to human and mouse gene names to prevent conversion to dates[3], it is uncertain whether such changes have propagated through to other species. To assess this, we downloaded all ...”

Gene name errors by year are described in more detail because this was the primary aim of this paper - to see if gene name errors are still an issue all these years after being first identified.

**R1P4. Calling a correlation coefficient of 0.589 a strong association is somewhat optimistic.**

Response: We have amended the wording of this section (page 13):

“A correlation analysis indicated a statistically significant association using the Pearson (p=0.0052, r=0.462,) and Spearman (p=1.95E-04; ρ=0.589) methods.”

**R1P5. This paper could be strengthened by offering more solutions to this problem, for example by emphasising using .csv rather than .txt format, and alternatives to spreadsheet software, such as RStudio.**

Response. In the discussion we have included a new information box titled “Tips to avoid gene name errors”. This contains practical steps researchers can take to minimise the risk of gene name errors. Box 1 is found on page 18-19.

**R1P6. A couple of minor points, it is possible to paste directly into Google Sheets without errors occurring.**

Response: The results shown in Table 1 represent the default software behaviour using the strings shown in the methods (now known as Box 2). We have confirmed that by default, Google Sheets does convert gene names to dates (See image below). Although SEPT7 was resistant to conversion, DEC1, OCT4 and MARCH3 were all converted to dates.

However as mentioned by Zeeberg and Colleagues in 2004, pre-formatting the cells as “Free text” is sufficient to prevent date conversion, therefore we have included the clarifying statement on page 5:

“The date conversion behaviour of Excel and Google Sheets could be circumvented by formatting the destination cells as “plain text” prior to pasting or typing. “

**R1P7. There appears to be an error with the automated reporting system, with four identical reports currently shown.**

Response: Thank you for identifying this error. We have made the necessary bug fixes, confirmed that the script is working as intended and updated the reports for the first 6 months of 2021. These reports are available at <http://ziemann-lab.net/public/gene_name_errors/>

## Reviewer #2

**I applaud the hard work of the authors. Opening several thousand files by hand is a heroic task that nobody envies them for. My hat is off!**

**I liked the analysis and the writing very much. I have only four minor points, the last one is optional:**

We thank reviewer 2 for their positive feedback on the paper.

**R2P1. Ordering table 4 by highest to lowest „Proportion of publications affected” makes grasping the results much easier.**

Response: In accordance with the suggestion, we have reordered Table 4 (page 10).

**R2P2. Show graph for the correlation between JIF and error rate and report R2. Such a graph is useful for visualizing the negative correlation of journal rank with reliability, consistent with similar graphs from other publications (see next point).**

Response: We have added a scatter plot (Figure 2 of new version). The corresponding text on page 13 has been amended for clarity:

“Next, we assessed whether there was any correlation between error proportion and the journal impact factor (JIF) for the set of 37 journals. A scatterplot of JIF and proportion of affected articles is shown in Figure 2. A correlation analysis indicated a statistically significant association using the Pearson (p=0.0052, r=0.462,) and Spearman (p=1.95E-04; ρ=0.589) methods.”

**R2P3. In the discussion (p14), the authors mention that the correlation of error rate with journal prestige may seem counterintuitive, but it is not only the 2016 analysis that their results are consistent with. See, e.g., https://doi.org/10.3389/fnhum.2018.00037 for a list of many other analyses that all suggest that work published in prestigious journals is more error prone than that of other publication venues. I fact, it would have been counter-intuitive had the authors found less errors in highly prestigious journals: the evidence predicts more errors in more prestigious journals.**

Response: We appreciate the reviewer’s point of view. We have mentioned this as a potential explanation for the observed JIF correlation, although on the balance of evidence we think the simpler explanation is that high JIF articles have more and larger supplementary files. See modified text on page 16:

“Another take-away from this study is that articles with supplementary Excel gene lists in highly reputable journals … more frequently contained gene name errors as compared to their counterparts with lower JIF scores. This may seem counterintuitive, but is consistent with previous analysis[2]. Although it has been suggested that articles in highly prestigious journals are of an inferior methodological quality[6], the simpler explanation is that the number and size of supplementary gene lists accompanying articles is the main contributor to this trend (although we have not examined this hypothesis quantitatively).”

**R2P4. The authors may consider inserting a few sentences into their discussion about a solution to this (and many other, related) problems with the data and code underlying our narratives: an infrastructure solution that integrates research data and code in a way that makes supplemental files unnecessary. Such a solution has been technically feasible for some years now and many people have suggested to use such solutions, e.g.:**

**https://journals.plos.org/plosbiology/article?id=10.1371/journal.pbio.3000117**

**http://www.theguardian.com/science/head-quarters/2015/may/12/will-traditional-science-journals-disappear**

**https://neuroneurotic.net/2015/07/17/revolutionise-the-publication-process/**

**https://micahallen.org/2015/03/20/short-post-my-science-fiction-vision-of-how-science-could-work-in-the-future/**

Response: We agree with the reviewer that supplementary files are a relic of a bygone era and could be wholly replaced by new publishing paradigms that allow for end-to-end reproducibility, data interactivity, and dynamic publications that update automatically when new data become available. That said, we think it would be better addressed in a separate article as the Discussion section is already very long.
